# How do Chinese people perceive their healthcare system? Inequality in public satisfaction with healthcare security

**DOI:** 10.3389/fpubh.2025.1529964

**Published:** 2025-05-01

**Authors:** Shengxian Bi, Huawei Tan, Dandan Guo, Xinyi Peng, Qijiao Yang, Tsung-han Weng, Yingchun Chen

**Affiliations:** ^1^School of Medicine and Health Management, Tongji Medical College, Huazhong University of Science and Technology, Wuhan, China; ^2^College of Education for the Future, Beijing Normal University, Zhuhai, China; ^3^Key Research Institute of Humanities & Social Sciences of Hubei Provincial Department of Education, Research Centre for Rural Health Service, Wuhan, China

**Keywords:** healthcare security, satisfaction, machine learning, social equity and trust, medical expenses

## Abstract

**Background:**

Satisfaction with healthcare security is a critical indicator of the effectiveness of health systems. Social equity and trust and the financial burden of healthcare are key socioeconomic factors that can significantly influence residents’ perceptions of healthcare security. This study aims to investigate the impact of social equity and trust and medical burden on satisfaction with healthcare security and to analyze their potential interaction mechanisms.

**Methods:**

Using data from 7,052 participants in the 2021 China General Social Survey, this study employed machine learning methods, including neural networks (NN), random forests (RF), and logistic regression (LR), to predict and classify satisfaction with healthcare security. Additionally, causal inference techniques were applied to identify the key determinants and estimate their effects on satisfaction levels, thereby uncovering the underlying causal mechanisms.

**Results:**

The predictive performance of the three machine learning methods was similar (*p* < 0.001). In the original models, the AUCs for LR, NN, and RF were 0.549, 0.563, and 0.534, respectively. After including factors related to social equity and trust, the AUCs for LR, NN, and RF improved to 0.633, 0.638, and 0.611, respectively. Among the three ML models, medical expenses and social equity and trust were identified as the most influential factors. Further causal analysis confirmed that higher levels of social equity and trust increased satisfaction with healthcare security, while a heavier medical burden reduced it. The analysis also revealed significant marginal effects, suggesting that the impact of social equity and trust varied across different levels.

**Conclusion:**

This study highlights the complex relationship between social equity and trust, medical burden, and satisfaction with healthcare security, offering theoretical support for understanding residents’ perceptions of healthcare security in various social contexts.

## 1 Introduction

Satisfaction with healthcare security refers to individuals’ overall assessment of the adequacy of their health system’s ability to provide medical care, social health insurance coverage and quality of services. It reflects public approval of national health policies and serves as a key indicator of the success of government health policies (1). While it shares similarities with related concepts such as patient satisfaction or satisfaction with the quality of care, it differs in scope. Patient satisfaction typically focuses on specific interactions between patients and healthcare providers, whereas satisfaction with healthcare security encompasses a wider range of social factors, including the accessibility, affordability and equity of the healthcare system (2, 3). Furthermore, unlike health equity, which measures disparities in health outcomes and healthcare accessibility, satisfaction with healthcare security specifically assesses the extent to which individuals believe their healthcare security needs are met, considering both healthcare services and the supporting social systems (4). In China, the importance of this concept is particularly pronounced. Since 2009, the Chinese government has implemented a series of profound and systematic healthcare reforms aimed at achieving universal healthcare coverage and effectively reducing the financial burden of healthcare on the public (5). These reforms have not only facilitated the popularization and equalization of healthcare services, but have also played a crucial role in addressing challenges such as urban–rural disparities and regional development imbalances. Against this backdrop, satisfaction with healthcare security has become an important measure of the effectiveness of these reforms and provides a unique perspective for understanding the public’s true attitudes toward the healthcare system.

Recent studies of satisfaction with healthcare security have mainly used traditional statistical methods, such as linear or logistic regression, which typically assume linear relationships between variables (6). However, while this linear assumption simplifies the analysis, it fails to capture the complex non-linear relationships and interactions between variables, leading to significant limitations. For example, in studies of patient satisfaction, researchers often focus on socio-economic status and type of health insurance as primary variables, but tend to overlook the combined effects of factors such as living conditions and quality of health care services (7). As a result, these studies do not fully reflect the mechanisms underlying satisfaction. Moreover, in China’s complex social context, where principles of fairness and trust are of paramount importance, their causal relationship with satisfaction with healthcare security remains insufficiently examined (8). Although machine learning techniques have recently shown promise in addressing high-dimensional data and non-linear relationships in satisfaction research, their explanatory capacity is constrained by a limited integration with theoretical or qualitative analysis (9–11). Therefore, applying ensemble machine learning methods to investigate the causal mechanisms underlying satisfaction with healthcare security offers both theoretical and practical value.

Based on the above, this study uses high-dimensional data from the 2021 China General Social Survey (CGSS) and employs an ensemble machine learning (ML) approach that integrates neural networks, random forests, and logistic regression. This study examines the core factors influencing public satisfaction with China’s healthcare system. It focuses on the relative importance of medical burden, and social equity and trust. Additionally, through theoretical analysis, we explore the causal relationships underlying satisfaction with healthcare security.

The following research questions have been proposed to address these objectives:

(1)   What are the key determinants of public satisfaction with healthcare security in China?
(2)   How do medical burden, social equity and trust interact to shape public satisfaction with healthcare security?
(3)   Can ensemble machine learning methods effectively capture and explain these relationships?

The following hypotheses are thus developed (12):

*H1*: Residents with similar medical conditions experience no significant differences in medical burden.

*H2*: Residents make rational assessments of their healthcare security status based on their personal circumstances.

*H3*: Residents’ satisfaction with healthcare security increases with perceived social equity and trust but decreases as medical burden rises.

This study contributes to the existing literature by integrating ensemble machine learning with theoretical analysis to establish causal relationships, thereby providing insights for improving public approval of China’s healthcare system.

## 2 Data and research methods

### 2.1 Data sources and preprocessing

#### 2.1.1 Data sources

The CGSS, initiated by the Chinese Academy of Social Sciences, is a large-scale continuous sample survey and an authoritative data source for studying work and employment, family and social life, and residents’ social attitudes (13). Conducted in accordance with the ethical principles of the Declaration of Helsinki, the CGSS ensures the protection of participants’ rights and privacy throughout the data collection process. The 2021 CGSS adopted a longitudinal study design and used a multi-stage stratified sampling method to ensure the representativeness of the sample across different regions and populations. The survey covered 31 provinces in China, including 151 counties, 604 administrative villages, and more than 10,000 households. A total of 10,136 questionnaires were collected in 2021. After excluding responses with missing key variables, invalid responses, or those responses inconsistent with the research objectives, 7,052 valid participants were retained for analysis.

#### 2.1.2 Data preprocessing

The outcome variable in this study is satisfaction with healthcare security, which refers to individuals’ overall evaluation of the healthcare services and social health insurance provided by the government (14). Respondents were asked, “How would you rate the medical security provided by the government to the people?” using a scale of 1 to 10, with the ratings reflecting their subjective perceptions of the adequacy and quality of healthcare coverage. While ‘medical security’ could also refer to aspects such as cybersecurity in healthcare (e.g., privacy), in this context it refers specifically to the provision of healthcare services and insurance. A score of 5 or below indicates dissatisfaction, while a score above 5 reflects satisfaction with the healthcare security system.

The four main categories of predictor variables are as follows:

(a)   Living conditions: region (15), age (16), education level (17), household registration (18), job or occupation, personal income, insurance expenses and subsistence allowance (19).
(b)   Insurance status: health insurance and critical illness insurance (20, 21).
(c)   Medical services: medical institution, clinic distance, doctor appointment time, waiting time, medical expenses and medical level (22–25).
(d)   Social equity and trust: trust in hospitals, fairness of medical treatment, and fairness of urban and rural rights (26, 27).

### 2.2 Research methods

#### 2.2.1 LR

Logistic regression is a generalized linear model commonly used for classification tasks, especially when the outcome variable is binary or ordinal (28). In this study, we employed logistic regression to predict the probability of satisfaction with healthcare security based on the explanatory variables. The model maps the linear combination of inputs to a logistic function, producing a probability value between 0 and 1. The parameters of the model are estimated using maximum likelihood estimation, which provides interpretable insights into the relationship between each predictor variable and the outcome (29). Although logistic regression assumes a linear relationship between the log odds and the predictors, it serves as a baseline model for comparison with more complex machine learning methods, highlighting the added value of capturing non-linear relationships in the data.

#### 2.2.2 NN

Artificial neural networks simulate the structure of biological neurons and typically consist of an input layer, one or more hidden layers, and an output layer (30, 31). In our study, the input layer receives data from the explanatory variables, including living conditions, insurance status, medical services, and social equity and trust. The hidden layers process these inputs through weighted links and activation functions, capturing the non-linear relationships between the predictors and the outcome. The output layer is responsible for predicting satisfaction with healthcare security. We used back-propagation (BP) neural networks, which learn the mapping relationships through forward signal propagation and adjust the network weights and thresholds through backward error propagation.

#### 2.2.3 RF

The core principle of the random forest algorithm is to combine weak classifiers into a strong classifier by aggregating multiple decision trees, thereby improving the accuracy and robustness of predictions (32, 33). This algorithm constructs decision trees by repeatedly performing random sampling with replacement on the training data set. Each tree is built using a subset of the explanatory variables, including living conditions, insurance status, medical services, and social equity and trust. The final prediction is determined by the majority vote of all trees, ensuring a balanced consideration of the various factors influencing satisfaction with healthcare security. This method is particularly effective in dealing with high-dimensional data and evaluating the relative importance of variables.

#### 2.2.4 Model evaluation

ML algorithms are typically evaluated using confusion matrices and model performance is assessed using the area under the receiver operating characteristic (ROC) curve (AUC) (34). The horizontal axis of the ROC curve represents the false positive (FP) rate, while the vertical axis represents the true positive (TP) rate. The true negative (TN) and false negative (FN) rates can also be derived from the curve. The combination of these four categories forms the test indicators for machine learning algorithms: accuracy, sensitivity, specificity, Youden’s index, positive predictive value (PPV), negative predictive value (NPV) and the balanced score (F1 score).

$\mathrm{Accuracy}=\frac{TP+TN}{TP+TN+FP+FN}$

$\mathrm{Sensitivity}=\frac{TP}{TP+FN}$

$\mathrm{Specificity}=\frac{TN}{TN+FP}$

${\mathrm{Youden}}^{’}s\;\mathrm{index}=\frac{TP}{TP+FN}+\frac{TN}{TN+FP}-1$

$PPV=\frac{TP}{TP+FP}$

$NPV=\frac{TN}{TN+FN}$

$\mathrm{F1 score}=\frac{2TP}{2TP+FP+FN}$

The values of the indicators range from 0 to 1, with a value close to 1 indicating a superior model prediction, and vice versa. Additionally, DeLong’s test is used to compare the performance of the ROC curves, with a *p*-value of less than 0.05 indicating a significant difference between the two curves (35).

The data were processed and analysed using Python 3.11, with missing values addressed, outliers (via the IQR method) identified, and inconsistencies resolved. Descriptive statistics were employed to summarize the key data characteristics. Chi-square tests were employed to explore associations between outcome and predictors. In addition, Python 3.11 was utilized for the development of ML models and the execution of DeLong’s test for the comparison of classifier performance. Statistical significance was assessed at a threshold of *p* < 0.05.

In addition to model evaluation, this study conducted a causal analysis of the key factors identified by machine learning. Based on the variable importance rankings from the LR, NN, and RF models, the most influential predictors of satisfaction with healthcare security were selected for further causal exploration (36). A mathematical schematic was developed using Microsoft Visio to illustrate the hypothesized causal relationships among key variables. By integrating empirical results with a structured causal framework, this approach enhances both the explanatory power and practical relevance of the findings.

## 3 Results

### 3.1 Characteristics of participants

Table 1 shows the analysis of the CGSS dataset, which includes 7,052 participants, 5,038 of whom expressed satisfaction with their healthcare security. A positive correlation was identified between the predictor variables and satisfaction with healthcare security. Specifically, higher levels of education and income, greater health insurance compensation, easier access to medical care, greater confidence in social equity and trust, and higher satisfaction with healthcare security were all associated. With the exception of medical institutions, the effects of all other variables on satisfaction with healthcare security were statistically significant (*p* < 0.05). Detailed descriptive statistics are provided in Supplementary Table 1.

**Table 1:** Characteristics of satisfaction with healthcare security.

Variables	Total	Healthcare security satisfaction		$\chi^{2}$	*p*-value
		Dissatisfied (*n* = 2014)	Satisfied (*n* = 5,038)		
Region					
Eastern	2,954 (41.9)	774 (26.2)	2,180 (73.8)	24.494	<0.001
Central	2022 (28.7)	659 (32.6)	1,363 (67.4)		
Western	2076 (29.4)	581 (28.0)	1,495 (72.0)		
Age					
18 ~ 44	3,190 (45.2)	716 (22.4)	2,474 (77.6)	108.28	<0.001
45 ~ 59	2,584 (36.6)	852 (33.0)	1732 (67.0)		
>60	1,278 (18.1)	446 (34.9)	832 (65.1)		
Educational level					
No schooling	431 (6.1)	154 (35.7)	277 (64.3)	257.401	<0.001
Basic education	3,583 (50.8)	1,250 (34.9)	2,333 (65.1)		
High school	1,418 (20.1)	388 (27.4)	1,030 (72.6)		
Higher education	1,620 (23.0)	222 (13.7)	1,398 (86.3)		
Household registration					
Agricultural household	4,460 (63.2)	1,457 (32.7)	3,003 (67.3)	101.707	<0.001
Non-agricultural household	1,401 (19.9)	288 (20.6)	1,113 (79.4)		
Resident household	1,191 (16.9)	269 (22.6)	922 (77.4)		
Job or occupation					
No	3,086 (43.8)	951 (30.8)	2,135 (69.2)	13.704	<0.001
Yes	3,966 (56.2)	1,063 (26.8)	2,903 (73.2)		
Personal income*					
Low	3,165 (44.9)	1,079 (34.1)	2086 (65.9)	136.242	<0.001
Medium	1997 (28.3)	580 (29.0)	1,417 (71.0)		
High	1890 (26.8)	355 (18.8)	1,535 (81.2)		
Insurance expenses*					
Low	3,854 (54.7)	1,155 (30.0)	2,699 (70.0)	8.692	0.013
Medium	2,834 (40.2)	756 (26.7)	2078 (73.3)		
High	364 (5.2)	103 (28.3)	261 (71.7)		
Subsistence allowance					
No	6,800 (96.4)	1962 (28.9)	4,838 (71.1)	8.043	0.005
Yes	252 (3.6)	52 (20.6)	200 (79.4)		
Health insurance					
No insurance	2,487 (35.3)	854 (34.3)	1,633 (65.7)	177.786	<0.001
Resident insurance	3,136 (44.5)	948 (30.2)	2,188 (69.8)		
Employee insurance	1,230 (17.4)	188 (15.3)	1,042 (84.7)		
Government-funded healthcare	199 (2.8)	24 (12.1)	175 (87.9)		
Critical illness insurance					
No	6,833 (96.9)	1968 (28.8)	4,865 (71.2)	6.322	0.012
Yes	219 (3.1)	46 (21.0)	173 (79.0)		
Medical institution					
No visit	1,677 (23.8)	471 (28.1)	1,206 (71.9)	2.908	0.406
Community hospital	1,248 (17.7)	373 (30.0)	875 (70.0)		
General hospital	3,588 (50.9)	1,005 (28.0)	2,583 (72.0)		
Private hospital	539 (7.6)	165 (30.6)	374 (69.4)		
Clinic distance					
No visit	1,677 (23.8)	471 (28.1)	1,206 (71.9)	118.328	<0.001
Very far	273 (3.9)	134 (49.1)	139 (50.9)		
Far	635 (9.0)	239 (37.6)	396 (62.4)		
Close	1868 (26.5)	562 (30.1)	1,306 (69.9)		
Very close	2,599 (36.9)	608 (23.4)	1991 (76.6)		
Doctor appointment time					
No visit	1,677 (23.8)	471 (28.1)	1,206 (71.9)	95.955	<0.001
Very long	308 (4.4)	146 (47.4)	162 (52.6)		
Long	739 (10.5)	275 (37.2)	464 (62.8)		
Short	1,466 (20.8)	388 (26.5)	1,078 (73.5)		
Very short	2,862 (40.6)	734 (25.6)	2,128 (74.4)		
Waiting time					
No visit	1,677 (23.8)	471 (28.1)	1,206 (71.9)	89.61	<0.001
Very long	447 (6.3)	200 (44.7)	247 (55.3)		
Long	1,078 (15.3)	363 (33.7)	715 (66.3)		
Short	1,553 (22.0)	398 (25.6)	1,155 (74.4)		
Very short	2,297 (32.6)	582 (25.3)	1715 (74.7)		
Medical expenses					
No visit	1,677 (23.8)	471 (28.1)	1,206 (71.9)	544.87	<0.001
Very expensive	1,001 (14.2)	531 (53.0)	470 (47.0)		
Expensive	1,607 (22.8)	565 (35.2)	1,042 (64.8)		
Cheap	1,464 (20.8)	269 (18.4)	1,195 (81.6)		
Very cheap	1,303 (18.5)	178 (13.7)	1,125 (86.3)		
Medical level					
No visit	1,677 (23.8)	471 (28.1)	1,206 (71.9)	188.307	<0.001
Very low	346 (4.9)	181 (52.3)	165 (47.7)		
Low	772 (10.9)	304 (39.4)	468 (60.6)		
High	1980 (28.1)	557 (28.1)	1,423 (71.9)		
Very high	2,277 (32.3)	501 (22.0)	1776 (78.0)		
Trust in hospitals					
Very distrustful	307 (4.4)	213 (69.4)	94 (30.6)	623.789	<0.001
Distrustful	1,089 (15.4)	535 (49.1)	554 (50.9)		
Trustful	3,808 (54.0)	955 (25.1)	2,853 (74.9)		
Very trustful	1848 (26.2)	311 (16.8)	1,537 (83.2)		
Fairness of medical treatment					
Very unfair	265 (3.8)	192 (72.5)	73 (27.5)	728.719	<0.001
Unfair	1,109 (15.7)	574 (51.8)	535 (48.2)		
Fair	4,252 (60.3)	1,054 (24.8)	3,198 (75.2)		
Very fair	1,426 (20.2)	194 (13.6)	1,232 (86.4)		
Fairness of urban and rural rights					
Very unfair	804 (11.4)	474 (59.0)	330 (41.0)	583.073	<0.001
Unfair	2,132 (30.2)	734 (34.4)	1,398 (65.6)		
Fair	3,356 (47.6)	708 (21.1)	2,648 (78.9)		
Very fair	760 (10.8)	98 (12.9)	662 (87.1)		

^*^Personal income and insurance expenses were categorized into three levels: Low, Medium, and High. Personal income was classified based on the per capita income of rural and urban residents. Insurance expenses were classified according to the per capita contributions to residents’ health insurance and employees’ health insurance.

### 3.2 Prediction results of ML

Table 2 shows the predictive performance of three ML algorithms used to predict satisfaction with healthcare security. In the original model, which included living conditions, insurance status and medical services as predictor variables, the AUC was 0.549 for LR, 0.563 for NN and 0.534 for RF. After adding social equity and trust as additional predictor variables, the models were re-estimated, resulting in significant improvements in predictive performance. The AUC increased to 0.633 for LR, 0.638 for NN and 0.611 for RF. Accuracy, sensitivity, specificity, Youden’s index, PPV, NPV and F1 score all showed significant improvement. DeLong’s test showed no significant differences in predictive performance between NN, RF and LR (*p* < 0.001), underscoring their robustness across diverse predictive frameworks.

**Table 2:** Performance metrics of ML.

Type	Model	AUC	*p*-value*	Accuracy	Sensitivity	Specificity	Youden’s index	PPV	NPV	F1 score
Original model	LR	0.549	Reference	0.719	0.936	0.162	0.098	0.742	0.496	0.828
	NN	0.563	<0.001	0.726	0.929	0.197	0.126	0.751	0.517	0.83
	RF	0.534	<0.001	0.728	0.979	0.091	0.069	0.733	0.625	0.838
Addition of social equity and trust	LR	0.633	Reference	0.755	0.909	0.357	0.266	0.784	0.605	0.842
	NN	0.638	<0.001	0.763	0.918	0.358	0.276	0.789	0.625	0.848
	RF	0.611	<0.001	0.757	0.945	0.276	0.221	0.769	0.665	0.848

^*^p-values were obtained from DeLong’s test, with the ROC curve of the logistic regression model used as the reference series.

### 3.3 Variable contribution analysis

**Figure 1:**
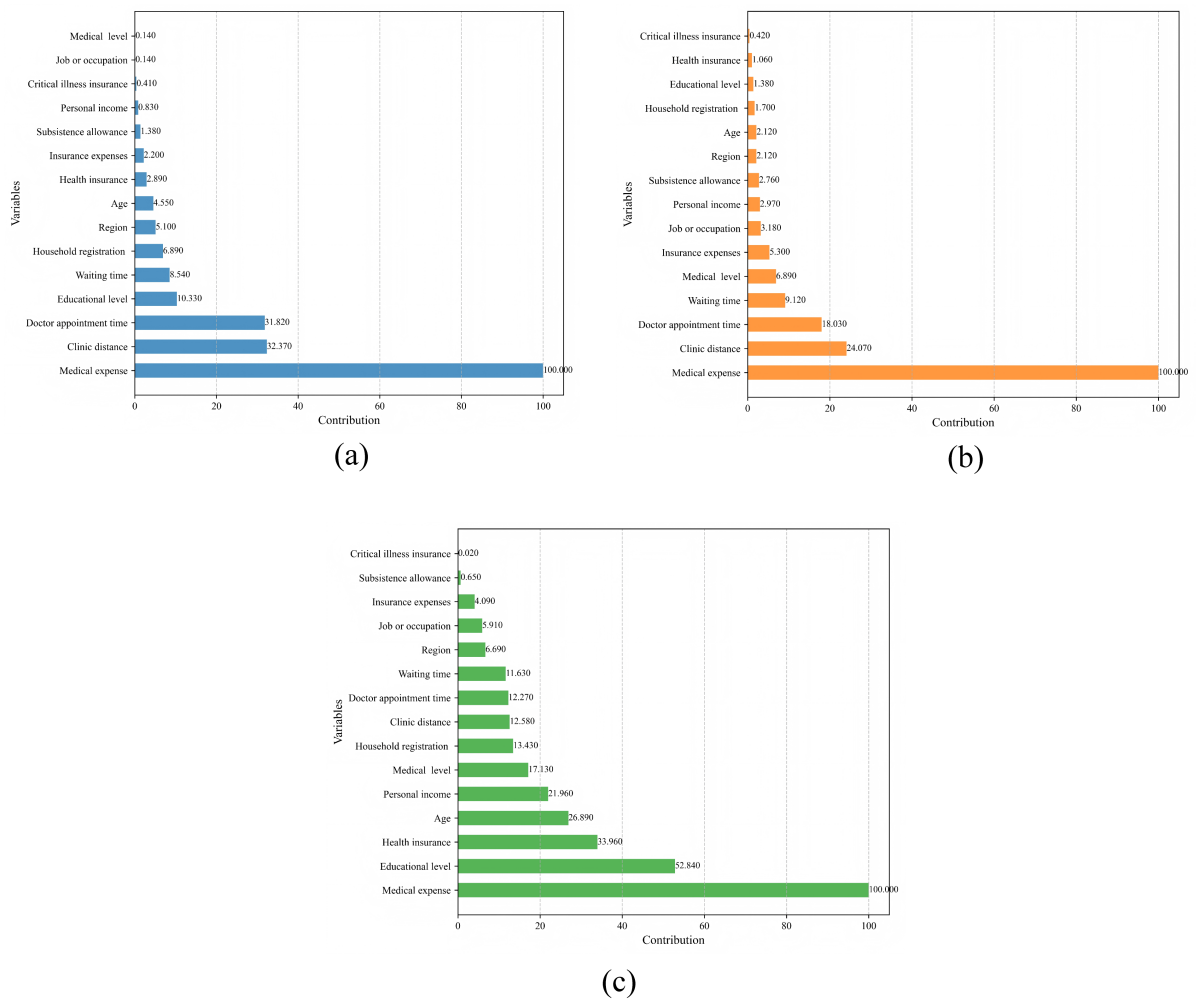
Variable contributions in original models. **(a)** LR; **(b)** NN; **(c)** RF.

**Figure 2:**
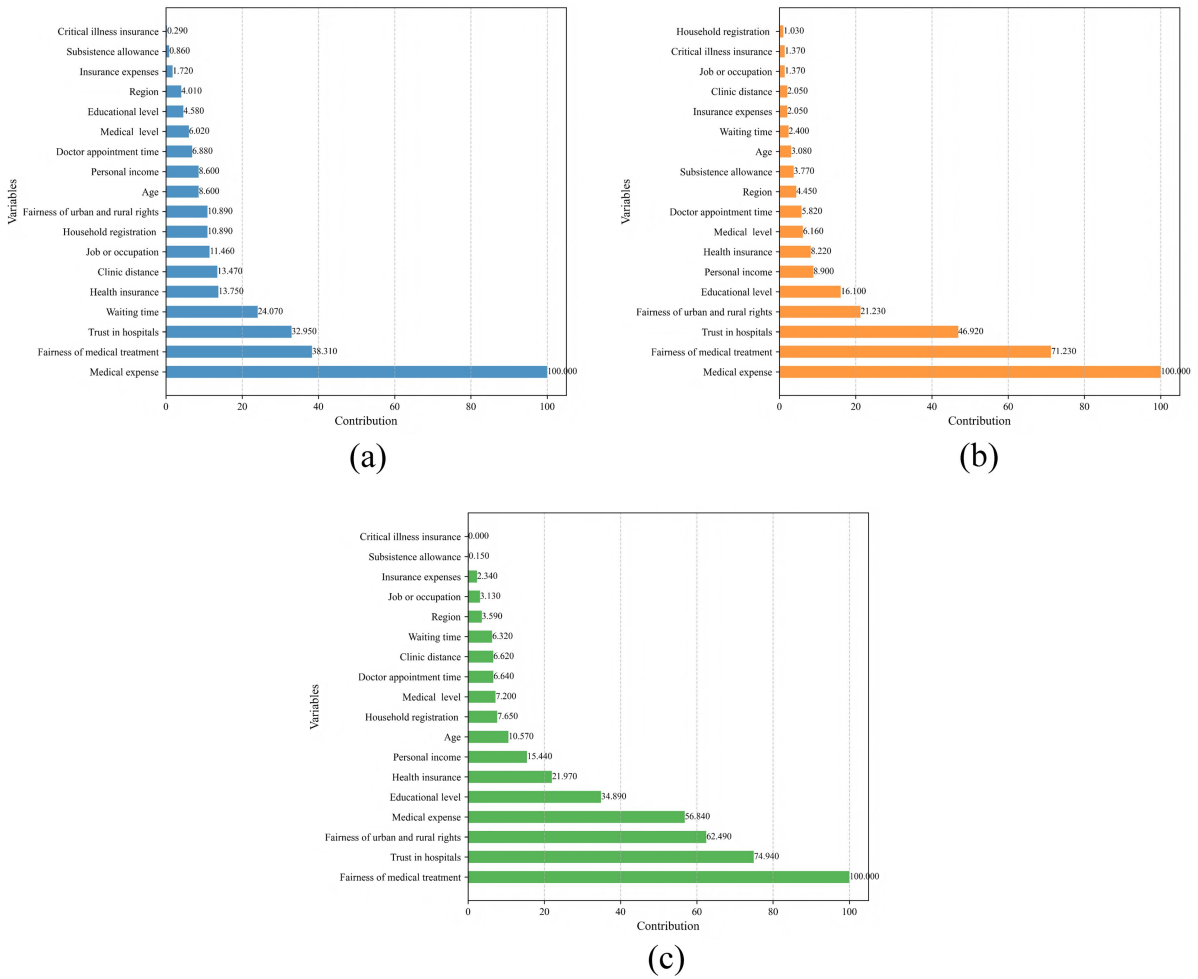
Variable contributions with social equity and trust. **(a)** LR; **(b)** NN; **(c)** RF.

Figures 1, 2 show the contributions of predictors in the original and enhanced models, respectively. Figures 1a–c present the variable contributions for LR, NN, and RF in the original model. In this model, medical expense was the most significant predictor in all three models, followed by clinic distance and doctor appointment time. Following the incorporation of social equity and trust as additional predictors, the models were re-estimated, as shown in Figures 2a–c. In the modified models, although medical expense remained the dominant factor in LR and RF, fairness of medical treatment and trust in hospitals became more prominent predictors. Notably, the relative importance of the predictors remained largely consistent across LR, NN and RF in both the original and modified models. This consistency suggests minimal heterogeneity in variable contributions across the different modeling approaches, further reinforcing the robustness of these predictors in explaining outcomes.

## 4 The relationship between social equity and trust, medical burden and satisfaction with healthcare security

**Figure 3:**
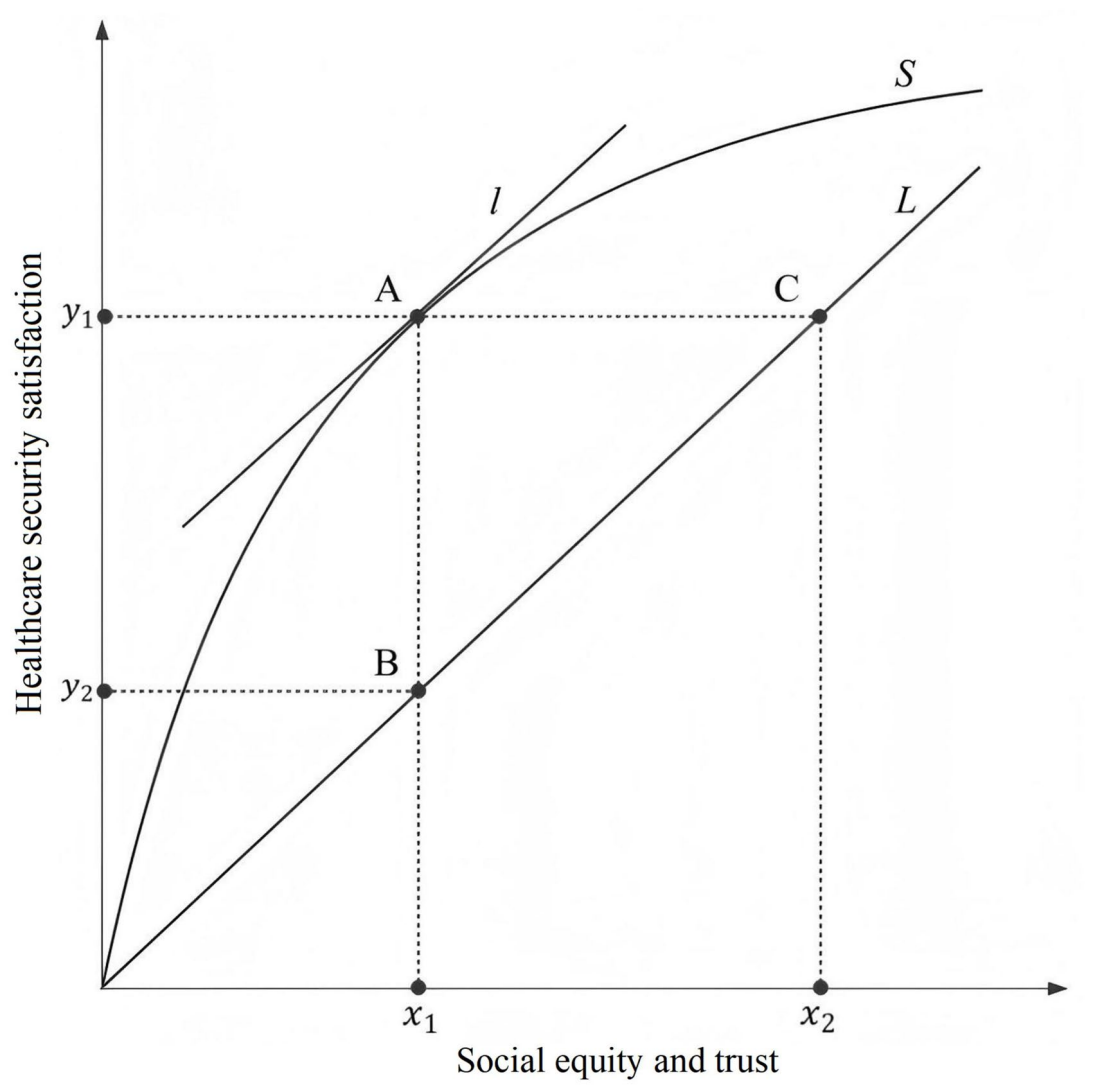
Schematic of function between social equity and trust and healthcare security satisfaction.

The ML algorithms identified that social equity and trust and medical expenses were the primary factors influencing participants’ satisfaction with healthcare security. In this section, we present a theoretical framework for understanding healthcare security satisfaction from a social equity and trust perspective. First, we describe the mechanism by which a single factor - social equity and trust - affects satisfaction with health security, as shown in Figure 3. In this figure, the horizontal axis represents the level of social equity and trust, while the vertical axis represents healthcare security satisfaction.

Ideally, satisfaction with healthcare security is a linear function of social equity and trust, represented by the ideal straight line L in the schematic. However, both satisfaction with healthcare security and social equity and trust are shaped by residents’ subjective feelings and influenced by psychological biases (37). As a result, satisfaction with healthcare security exhibits different growth patterns under varying degrees of social equity and trust, as shown by the actual curve S in the schematic. If the line L is the tangent at point A on the curve S, then the slope K_A_ = K_L_. Point A is called the social equity and trust threshold point, with the horizontal coordinate corresponding to point A representing the social equity and trust threshold. Let point D be an arbitrary point assumed to the left of curve S at point A, and point E an arbitrary point assumed to the right of curve S at point A. It is easy to see that the relationship between the slopes of the tangents at these three points is K_D_ > K_A_ > K_E_. In other words, the slope of the tangent on curve S decreases as social equity and trust increases. This suggests that, all other things being equal, the rate of increase in satisfaction with healthcare security decreases as the level of social equity and trust increases by the same proportion (see Figure 3). This phenomenon is known as the marginal effect of social equity and trust. The marginal effect of social equity and trust indicates that in the early stages of increasing social equity and trust, the growth in healthcare security satisfaction is much higher than the ideal policy level. However, once the effect of social equity and trust reaches the ideal policy level, the growth trend in healthcare security satisfaction slows down. To illustrate this, we refer to China’s rural health insurance system in the 1960s and 1970s and the current health insurance system (38). When the overall level of social development was low, modest investment in health insurance led to high satisfaction among residents (39). However, as the economic level improved significantly, despite substantial increases in health insurance premiums each year, the growth trend in residents’ satisfaction with healthcare security became less pronounced (40).

In the schematic, we refer to the horizontal difference between the actual curve and the ideal straight line as the social equity and trust deviation, and the vertical difference as the satisfaction deviation. These deviations reflect the impact of policy implementation. Assuming the existence of multiple curves, where each curve represents the outcome of a different policy, we found that a large deviation indicates a strong policy stimulus but challenges in ensuring long-term policy sustainability. In contrast, a small deviation indicates a weak policy stimulus and slow progress toward health equity. The social equity and trust deviation and satisfaction deviation suggest that a robust and sustainable policy is the optimal choice to promote health equity. In addition, it is important to consider the conditions at both ends of the curve. As the degree of social equity and trust approaches infinity, satisfaction with healthcare security will asymptotically approach 1, but will never reach 1 due to the law of diminishing marginal benefits (41). If the degree of social equity and trust is zero, it will be difficult for residents to make a rational assessment of their health security status, which contradicts the assumptions made in the model.

**Figure 4:**
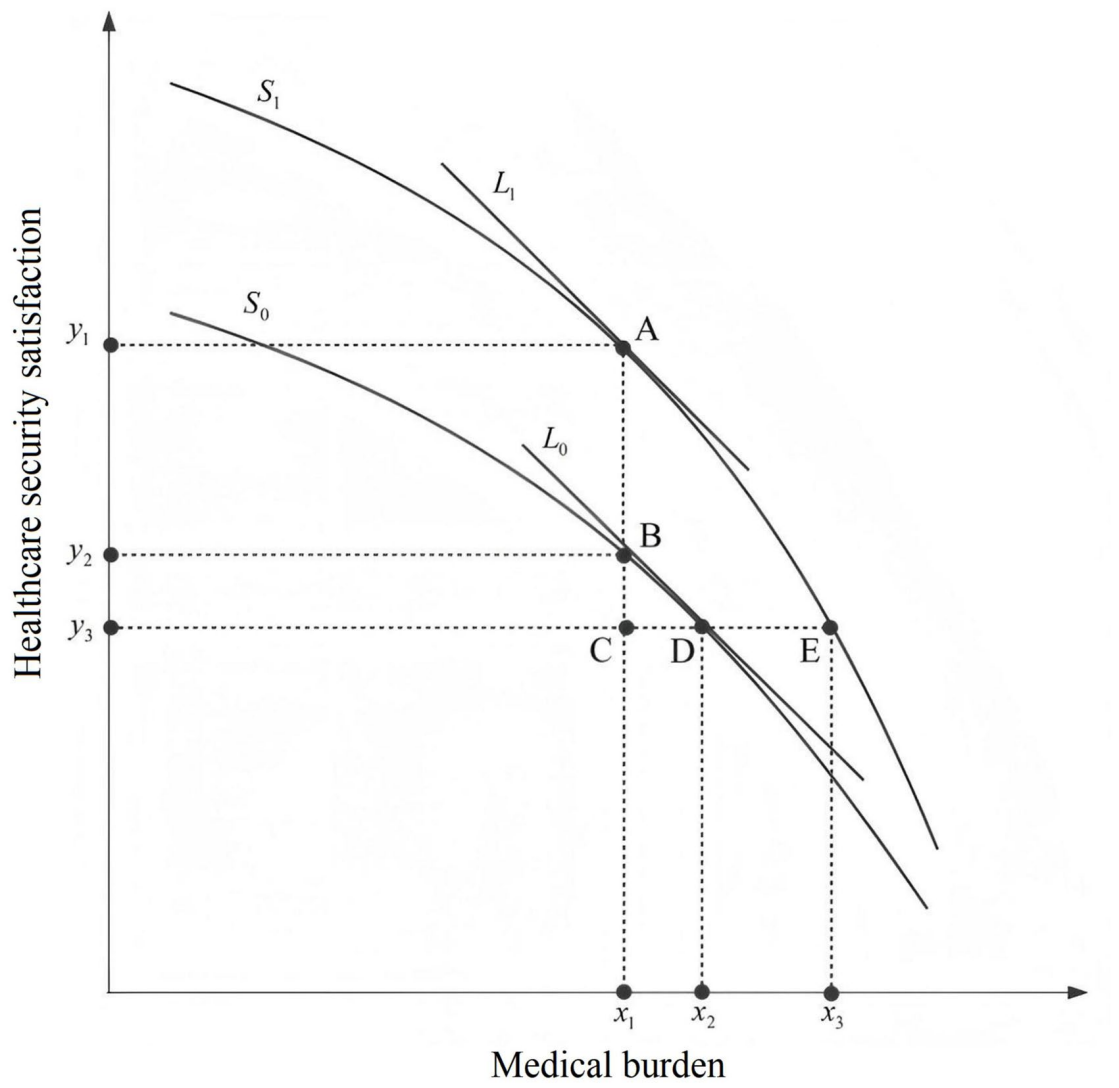
Schematic of function between medical burden, social equity and trust, and healthcare security satisfaction.

Building on the previous analysis of how social equity and trust influence satisfaction with healthcare security, we introduced the medical burden as an additional factor. This allowed us to further examine its effect on satisfaction under varying levels of social equity and trust (see Figure 4). In Figure 4, the horizontal axis represents the medical burden. This measure considers residents’ income relative to their medical expenses, providing a comprehensive indication of their ability to afford healthcare. The vertical axis represents satisfaction with healthcare security. S_1_ and S_0_ are the perceptual difference curves depicting residents’ healthcare security satisfaction at high and low levels of social equity and trust, respectively, while L_1_ and L_0_ are the tangents to the corresponding curves with slopes K_1_ = K_0_.

Ideally, S_1_ and S_0_ would represent straight lines for healthcare security satisfaction, reflecting changes in medical burden. However, due to residents’ psychological affordability limits, the downward trend in healthcare security satisfaction remains subtle until the medical burden reaches the threshold of affordable health expenditure (hereafter referred to as the burden threshold). Beyond this point, satisfaction with healthcare security declines rapidly as the medical burden exceeds residents’ affordability range. The burden threshold also varied due to differences in residents’ perceptions of medical burden across varying levels of equity. This effect is particularly noticeable among residents with high perceived equity (42). In contrast, residents with low perceived equity tend to have lower expectations of their health security status, resulting in a relatively higher burden threshold. In the schematic, if point A represents the burden threshold on curve S_1_, then ideally point B with the same medical burden index would correspond to the burden threshold on curve S_0_. However, the slope of the tangent at point B is less than that at point A for the reasons given above. If point D has the same slope as point A, it would represent the burden threshold at level S_0_. The horizontal distance between points x_2_ and x_1_ can then be interpreted as the burden threshold deviation. Similarly, the vertical difference between y_1_ and y_3_ reflected the satisfaction threshold deviation between the two threshold points. These deviations reflect differences in affordability and satisfaction at the inflection point of the healthcare security satisfaction curve. Larger deviations suggest greater disparities in social equity and trust. The deviation in the load threshold from point B to point D on S_0_ is captured by the vertical difference y_2_-y_3_, which we call the equity deviation. This represents the satisfaction gap between the theoretical and actual inflection points of the curve. To further explain the different downward trends of the curves before and after the burden threshold, we combine the two curves. Before the load threshold is reached, the slope K_B_ of the tangent at point B is less than the slope K_BD_ of the straight line BD, and point B on the straight line BD has the same slope as point D. At point D, we find that the slope of the straight line BD and the tangent both satisfy the relationship K_BD_ < K_0_. We have used K_BD_ as an intermediary to establish the relationship K_B_ < K_0_ between the tangents at points B and D. Similarly, for the trend of the curve after the load threshold is reached, we examine the transfer effect of line AE. Using this, we derived the slope K_1_ of the tangent at point A. We then find that the slope K_E_ of the tangent at point E satisfied the relationship K_1_ < K_E_.

In the discussion above, we primarily considered the case of the load threshold. Next, we held either the *x*-axis or the *y*-axis constant to further explore the difference between the two curves. When the medical burden was fixed at x_1_, the vertical difference between S_1_ and S_0_ was defined as the satisfaction gap. Conversely, when satisfaction with healthcare security was fixed at y_3_, the horizontal difference between S_1_ and S_0_ was defined as the burden gap. Both gaps measure the degree of variation in social equity and trust. Finally, it is necessary to examine the endpoints of the curve. When the medical burden was close to zero, residents’ satisfaction with healthcare security reflected a strong sense of access to high-quality medical and health services. This was particularly evident among residents covered by government-funded health insurance. In contrast, when satisfaction with healthcare security was close to zero, the situation was markedly different. At this point, the severity of illness and the cost of diagnosis and treatment far exceeded the household’s financial capacity. In some cases, residents were entering the end-of-life stage. Under such circumstances, it became difficult for them to make rational assessments or judgments about their medical security status (43). These two situations contradicted our assumptions that residents experience no significant differences in medical burden (H1) and that they make rational assessments of their healthcare security status (H2). Therefore, we did not fully analyse the two ends of the curve. Taken together, Figures 3, 4 provide empirical support for Hypothesis 3, while partially disproving Hypotheses 1 and 2 due to the deviations observed at both extremes of the curve.

## 5 Discussion

This study extends the empirical analysis by using ML algorithms to examine the causal relationship between outcome equity and opportunity equity in healthcare security. Using the latest data from the CGSS, with satisfaction with healthcare security serving as a comprehensive indicator, this research elucidates how social and economic factors shape individuals’ perceptions of healthcare security. By integrating predictive modeling with causal inference, this approach not only strengthens the analytical rigour of the research, but also enhances the policy relevance of its findings.

First, social equity and trust and healthcare security satisfaction are mutually influential. Our findings indicate that social equity and trust is a significant contributing factor to healthcare security satisfaction, while healthcare security satisfaction itself is a crucial component of social equity. The relationship between these two variables is not a simple positive linear one, but has a non-linear marginal effect. Specifically, as the level of social equity and trust increases, the rate of improvement in healthcare security satisfaction slows down. Conversely, when healthcare security satisfaction improves, social equity and trust tends to increase more rapidly. This result suggests that improvements in social policies not only enhance healthcare security satisfaction but also foster broader social equity and trust (44).

Secondly, medical burden is the most significant factor influencing satisfaction with healthcare security. As the medical burden increases, residents’ satisfaction with healthcare security gradually declines. By analyzing the inflection point of the satisfaction curve, we introduce a new definition of catastrophic healthcare expenditure: when medical burden reaches a certain threshold, residents’ satisfaction with healthcare security drops sharply (45). This critical point is not only related to the financial burden of health care costs, but also to the psychological capacity of residents to bear such burdens. In other words, catastrophic health expenditure reflects not only an economic burden but also the psychological and emotional resilience of residents.

Third, regarding the satisfaction curve for healthcare security under varying levels of equity, we find that it does not resemble the indifference curves found in economics. In areas with higher health equity, the critical threshold for the burden on residents is lower, and satisfaction with health care tends to decline more easily. International comparisons support this finding: residents in developed countries may protest more strongly against cuts in health services, while in regions such as sub-Saharan Africa, despite lower levels of health care, residents may react less strongly to cuts (46, 47). This disparity suggests that satisfaction with health care is shaped not only by economic factors, but also by the social context and cultural expectations of the population (48). Future CGSS data can be used to test this model by examining non-linear relationships between perceived equity and satisfaction, and identifying potential threshold effects across regions and social groups.

There are several limitations to this study. First, the data used in this study are from a large survey database in China, where healthcare data are subject to recall bias due to self-reporting, which may lead to inaccuracies. Additionally, this study does not analyze the potential mediating relationships between social equity and trust, satisfaction with healthcare security and medical burden, which limits our understanding of the complex interactions among these variables. Furthermore, the schematic illustrations of healthcare security satisfaction in this study are not based on specific mathematical equations estimated from the data, but rather serve as conceptual visualizations of possible functional patterns to support causal reasoning. While such diagrams help in illustrating theoretical mechanisms, they do not provide definitive empirical evidence to confirm or reject the proposed hypotheses. Future research could address these limitations by using more comprehensive datasets and exploring the mediating factors between key variables to gain deeper insights into their interrelationships.

## 6 Conclusion

This study utilizes the CGSS database and ML algorithms to predict and classify healthcare security satisfaction, aiming to identify its key determinants and explore the underlying mechanisms through causal analysis. The findings indicate that social equity and trust and medical burden are core factors influencing satisfaction with healthcare security. An increase in social equity and trust is positively correlated with higher satisfaction with healthcare security, whereas an increase in medical burden significantly diminishes it. The study also reveals the marginal effects between social equity and trust and healthcare satisfaction: at higher levels of social equity and trust, the rate of improvement in satisfaction with healthcare security decelerates; conversely, greater satisfaction with healthcare security accelerates the rise in social equity and trust. These findings effectively explain the mechanisms that shape health satisfaction in different social contexts and provide valuable insights for improving the equity of health policies worldwide. To this end, policymakers should implement targeted financial assistance programmes to reduce the burden of health care and prevent excessive out-of-pocket costs for low-income populations. In addition, increasing the transparency of health care governance and improving the efficiency of services can further enhance public trust and satisfaction.

## Data Availability

The datasets presented in this study can be found in online repositories. The names of the repository/repositories and accession number(s) can be found at: Chinese General Social Survey (cgss.ruc.edu.cn).
