# Supplementary material for: How do Chinese people perceive their healthcare system? Inequality in public satisfaction with healthcare security
Source: Front Public Health. 2025 May 1;13:1529964. doi: 10.3389/fpubh.2025.1529964 (PMC12078328; doi:10.3389/fpubh.2025.1529964)
Supplement: Supplementary file 1 [file Table_1.docx]

**Supplementary Table 1: Descriptive statistics of the dataset**

| Variables | Mean | SD | Min | Q1 | Med | Q3 | Max |
| --- | --- | --- | --- | --- | --- | --- | --- |
| Region | | | | | | | |
| Eastern | 7.178 | 2.527 | 1 | 5 | 8 | 10 | 10 |
| Central | 6.813 | 2.671 | 1 | 5 | 7 | 9 | 10 |
| Western | 7.169 | 2.610 | 1 | 5 | 8 | 10 | 10 |
| Age | | | | | | | |
| 18~44 | 7.382 | 2.305 | 1 | 6 | 8 | 10 | 10 |
| 45~59 | 6.839 | 2.775 | 1 | 5 | 7.5 | 10 | 10 |
| >60 | 6.763 | 2.828 | 1 | 5 | 7 | 10 | 10 |
| Educational level | | | | | | | |
| No schooling | 6.872 | 3.041 | 1 | 5 | 8 | 10 | 10 |
| Basic education | 6.763 | 2.773 | 1 | 5 | 7 | 10 | 10 |
| High school | 7.157 | 2.490 | 1 | 5 | 8 | 9.75 | 10 |
| Higher education | 7.729 | 1.961 | 1 | 7 | 8 | 9 | 10 |
| Household registration | | | | | | | |
| Agricultural household | 6.859 | 2.713 | 1 | 5 | 7 | 10 | 10 |
| Non-agricultural household | 7.508 | 2.312 | 1 | 6 | 8 | 10 | 10 |
| Resident household | 7.350 | 2.378 | 1 | 6 | 8 | 9 | 10 |
| Job or occupation | | | | | | | |
| No | 6.928 | 2.613 | 1 | 5 | 8 | 9 | 10 |
| Yes | 7.182 | 2.582 | 1 | 5 | 8 | 10 | 10 |
| Personal income | | | | | | | |
| Low | 6.779 | 2.747 | 1 | 5 | 7 | 9 | 10 |
| Medium | 7.101 | 2.630 | 1 | 5 | 8 | 10 | 10 |
| High | 7.528 | 2.214 | 1 | 6 | 8 | 10 | 10 |
| Insurance expenses | | | | | | | |
| Low | 7.010 | 2.682 | 1 | 5 | 8 | 10 | 10 |
| Medium | 7.155 | 2.511 | 1 | 5 | 8 | 9 | 10 |
| High | 7.060 | 2.342 | 1 | 5 | 7 | 9 | 10 |
| Subsistence allowance | | | | | | | |
| No | 7.055 | 2.602 | 1 | 5 | 8 | 10 | 10 |
| Yes | 7.508 | 2.470 | 1 | 6 | 8 | 10 | 10 |
| Health insurance | | | | | | | |
| No insurance | 6.716 | 2.735 | 1 | 5 | 7 | 9 | 10 |
| Resident insurance | 7.006 | 2.631 | 1 | 5 | 8 | 10 | 10 |
| Employee insurance | 7.774 | 2.087 | 1 | 7 | 8 | 10 | 10 |
| Government-funded healthcare | 8.171 | 1.988 | 1 | 7 | 8 | 10 | 10 |
| Critical illness insurance | | | | | | | |
| No | 7.054 | 2.601 | 1 | 5 | 8 | 10 | 10 |
| Yes | 7.580 | 2.460 | 1 | 6 | 8 | 10 | 10 |
| Medical institution | | | | | | | |
| No visit | 7.206 | 2.713 | 1 | 5 | 8 | 10 | 10 |
| Community hospital | 6.988 | 2.552 | 1 | 5 | 8 | 9 | 10 |
| General hospital | 7.052 | 2.554 | 1 | 5 | 8 | 9 | 10 |
| Private hospital | 6.965 | 2.625 | 1 | 5 | 8 | 9 | 10 |
| Clinic distance | | | | | | | |
| No visit | 7.206 | 2.713 | 1 | 5 | 8 | 10 | 10 |
| Very far | 5.923 | 3.129 | 1 | 4 | 6 | 8 | 10 |
| Far | 6.493 | 2.648 | 1 | 5 | 7 | 8 | 10 |
| Close | 6.941 | 2.537 | 1 | 5 | 8 | 9 | 10 |
| Very close | 7.339 | 2.429 | 1 | 6 | 8 | 10 | 10 |
| Doctor appointment time | | | | | | | |
| No visit | 7.206 | 2.713 | 1 | 5 | 8 | 10 | 10 |
| Very long | 5.886 | 2.913 | 1 | 4 | 6 | 8 | 10 |
| Long | 6.510 | 2.563 | 1 | 5 | 7 | 8 | 10 |
| Short | 7.087 | 2.431 | 1 | 5 | 8 | 9 | 10 |
| Very short | 7.255 | 2.534 | 1 | 5 | 8 | 10 | 10 |
| Waiting time | | | | | | | |
| No visit | 7.206 | 2.713 | 1 | 5 | 8 | 10 | 10 |
| Very long | 6.000 | 2.878 | 1 | 4.5 | 6 | 8 | 10 |
| Long | 6.713 | 2.473 | 1 | 5 | 7 | 8 | 10 |
| Short | 7.158 | 2.434 | 1 | 5 | 8 | 9 | 10 |
| Very short | 7.289 | 2.556 | 1 | 5 | 8 | 10 | 10 |
| Medical expenses | | | | | | | |
| No visit | 7.206 | 2.713 | 1 | 5 | 8 | 10 | 10 |
| Very expensive | 5.563 | 2.946 | 1 | 3 | 5 | 8 | 10 |
| Expensive | 6.601 | 2.466 | 1 | 5 | 7 | 8 | 10 |
| Cheap | 7.584 | 2.173 | 1 | 6 | 8 | 10 | 10 |
| Very cheap | 8.057 | 2.076 | 1 | 7 | 8 | 10 | 10 |
| Medical level | | | | | | | |
| No visit | 7.206 | 2.713 | 1 | 5 | 8 | 10 | 10 |
| Very low | 5.546 | 3.067 | 1 | 3 | 5 | 8 | 10 |
| Low | 6.242 | 2.547 | 1 | 5 | 6 | 8 | 10 |
| High | 7.005 | 2.429 | 1 | 5 | 7 | 9 | 10 |
| Very high | 7.541 | 2.433 | 1 | 6 | 8 | 10 | 10 |
| Trust in hospitals | | | | | | | |
| Very distrustful | 4.573 | 2.942 | 1 | 1 | 5 | 7 | 10 |
| Distrustful | 5.848 | 2.735 | 1 | 4 | 6 | 8 | 10 |
| Trustful | 7.141 | 2.348 | 1 | 5 | 8 | 9 | 10 |
| Very trustful | 8.061 | 2.379 | 1 | 7 | 9 | 10 | 10 |
| Fairness of medical treatment | | | | | | | |
| Very unfair | 4.109 | 3.060 | 1 | 1 | 4 | 6 | 10 |
| Unfair | 5.625 | 2.644 | 1 | 4 | 5 | 8 | 10 |
| Fair | 7.235 | 2.326 | 1 | 5 | 8 | 9 | 10 |
| Very fair | 8.255 | 2.316 | 1 | 7 | 9 | 10 | 10 |
| Fairness of urban and rural rights | | | | | | | |
| Very unfair | 5.157 | 3.001 | 1 | 2 | 5 | 8 | 10 |
| Unfair | 6.623 | 2.492 | 1 | 5 | 7 | 8 | 10 |
| Fair | 7.493 | 2.294 | 1 | 6 | 8 | 10 | 10 |
| Very fair | 8.487 | 2.296 | 1 | 8 | 10 | 10 | 10 |
